# Influence of Quadrupolar Molecular Transitions within Plasmonic Cavities

**DOI:** 10.1021/acsnano.4c01368

**Published:** 2024-05-24

**Authors:** Junyang Huang, Oluwafemi S. Ojambati, Clàudia Climent, Alvaro Cuartero-Gonzalez, Eoin Elliott, Johannes Feist, Antonio I. Fernández-Domínguez, Jeremy J. Baumberg

**Affiliations:** † NanoPhotonics Centre, Cavendish Laboratory, Department of Physics, JJ Thompson Avenue, University of Cambridge, Cambridge CB3 0HE, U.K.; ‡ Departamento de Física Teórica de la Materia Condensada and Condensed Matter Physics Center (IFIMAC), Universidad Autónoma de Madrid, Madrid E-28049, Spain; § Department of Chemistry, University of Pennsylvania, Philadelphia, Pennsylvania 19104, United States; ∥ Mechanical Engineering Department, ICAI, Universidad Pontificia Comillas, Madrid 28015, Spain

**Keywords:** plasmonics, quadrupolar transition, field gradient, molecular photoluminescence, nanoparticle

## Abstract

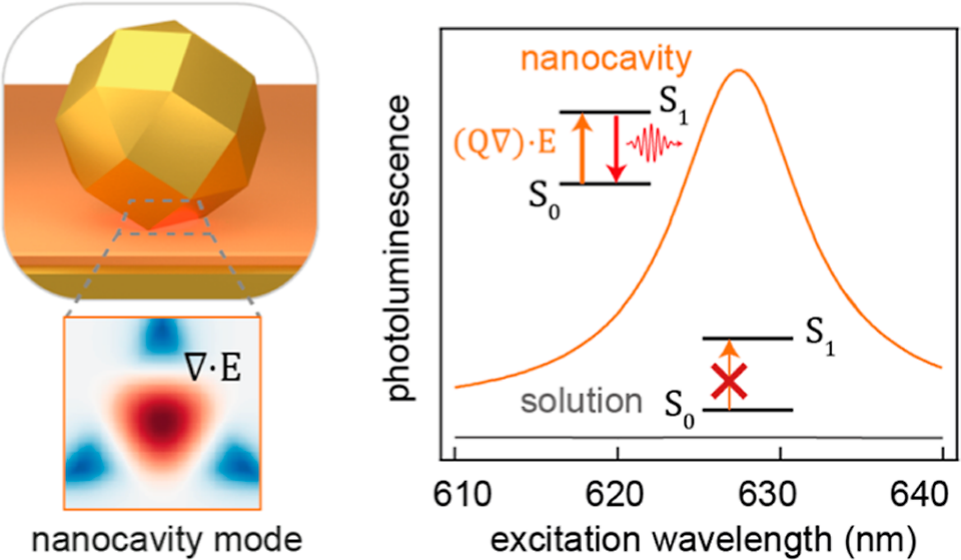

Optical nanocavities have revolutionized the manipulation of radiative properties of molecular and semiconductor emitters. Here, we investigate the amplified photoluminescence arising from exciting a dark transition of β-carotene molecules embedded within plasmonic nanocavities. Integrating a molecular monolayer into nanoparticle-on-mirror nanostructures unveils enhancements surpassing 4 orders of magnitude in the initially light-forbidden excitation. Such pronounced enhancements transcend conventional dipolar mechanisms, underscoring the presence of alternative enhancement pathways. Notably, Fourier-plane scattering spectroscopy shows that the photoluminescence excitation resonance aligns with a higher-order plasmonic cavity mode, which supports strong field gradients. Combining quantum chemistry calculations with electromagnetic simulations reveals an important interplay between the Franck–Condon quadrupole and Herzberg–Teller dipole contributions in governing the absorption characteristics of this dark transition. In contrast to free space, the quadrupole moment plays a significant role in photoluminescence enhancement within nanoparticle-on-mirror cavities. These findings provide an approach to access optically inactive transitions, promising advancements in spectroscopy and sensing applications.

## Introduction

Optical nanocavities have been extensively explored to modify the radiative properties of molecular and semiconductor emitters.^1−8^ Plasmonic nanoparticles of noble metals support collective oscillations of conduction electrons known as plasmons, which can confine optical fields to mode volumes far below the diffraction limit (<100 nm^3^), yielding field enhancements exceeding 500.^9^ This strong concentration of optical fields in plasmonic nanocavities can routinely boost the interaction of quantum emitters with light, resulting in Purcell-accelerated spontaneous emission rates,^6^ modified charge relaxation pathways,^10,11^ enhanced fluorescence,^12,13^ highly efficient nonlinear optical effects,^14^ and polaritonic phenomena.^7,15,16^

In free space, optical fields are spatially uniform over the size of quantum emitters, and their interaction with light is completely governed by electrical-dipole-active excitations. However, higher-order multipolar transitions in quantum emitters become relevant when strong field gradients are present in their photonic environment.^17−20^ Recent studies have shown how field gradients unreachable by conventional optics (e.g., optical evanescent fields or tweezing) become accessible through tightly confined plasmonic modes supported by nanocavities.^21−25^ In such nanostructures, the modal frequencies, radiative efficiencies, and electric field spatial characteristics can be tuned via multiple design parameters.^9,23,26^ This makes them ideally suited for the excitation and probing of far-field-forbidden excitations in quantum emitters.^27−29^ However, these theoretical predictions have yet to be experimentally demonstrated.

Here, we study the enhanced photoluminescence (PL) from a dark transition of β-carotene by integrating a molecular ensemble into nanoparticle-on-mirror (NPoM) plasmonic nanocavities. Our investigation begins with the characterization of various plasmonic modes supported by these NPoM structures, employing Fourier-plane-based energy-momentum spectroscopy to delineate their scattering properties. Central to our experimental design is the assessment of absorption and radiative efficiencies of molecular transitions through PL analysis, in both the presence and absence of plasmonic nanocavities. We employ two excitation wavelengths specifically selected to excite distinct absorption pathways that are governed by bright and dark transitions. We demonstrate that by coupling the molecules to NPoM plasmonic modes, the contribution from the originally light-forbidden excitation is enhanced by more than 4 orders of magnitude. Finally, we present a numerical analysis combining quantum chemistry and classical electromagnetic calculations which reveal that the β-carotene dark transition observed in PL spectra has hybrid Franck–Condon quadrupole^30^ and Herzberg–Teller dipole^31,32^ character. Our theoretical results indicate that the latter governs the radiative characteristics of the molecules, while both the dipole and quadrupole transition moments similarly contribute to the optical absorption. Our results provide an encouraging approach to directly access dark electronic transitions, such as higher-order multipole moments or symmetry-forbidden dipoles, potentially applicable in spectroscopic sensing, improving molecular light emitting diodes, and molecular spintronics.

## Results and Discussion

**Figure 1:**
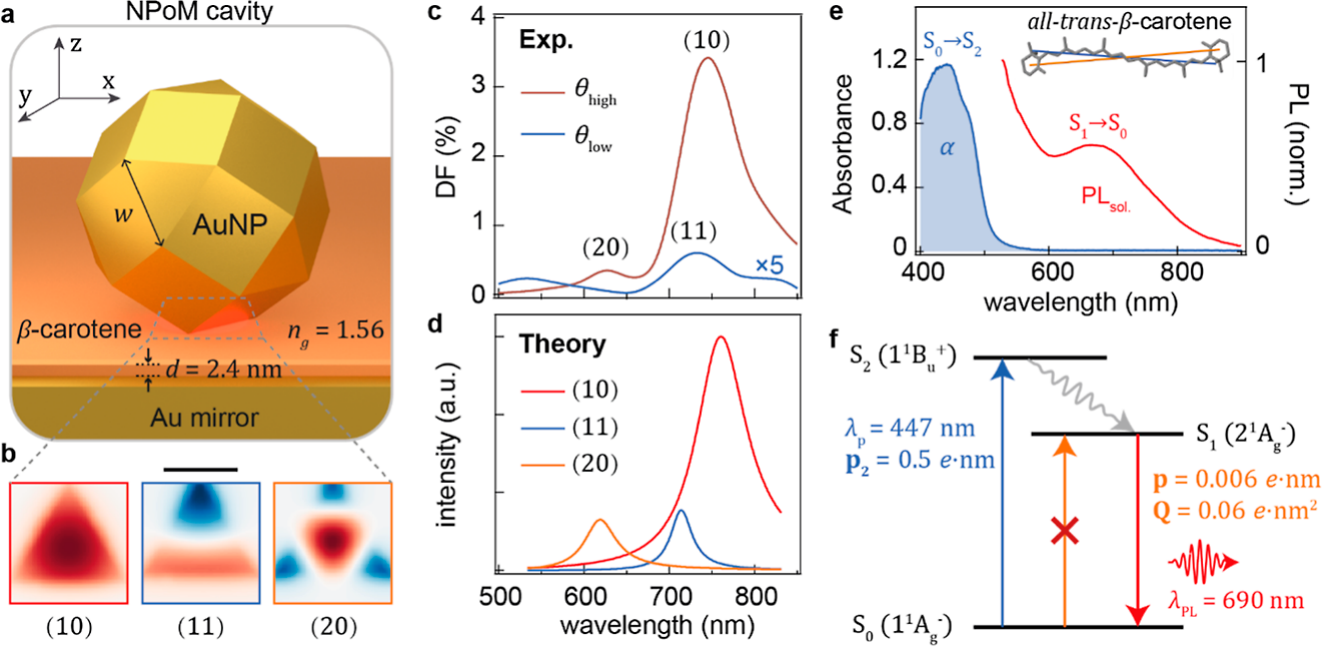
Plasmonic modes of NPoM plasmonic cavities and vibronic transitions of β-carotene. (a) Rhombicuboctahedral nanocavity geometry with β-carotene molecular spacer (orange) between the Au mirror and Au nanoparticle (with triangular facet face-down). (b) Normalized vertical component of optical field at the midplane of the gap for (10), (11), and (20) plasmonic modes (scale bar is 20 nm). (c) Experimental dark-field scattering spectra at high (θ_high_) and low (θ_low_) collection angles (from the mirror normal) of β-carotene-NPoMs, revealing modes as labeled. (d) Simulated resonances for the three plasmonic modes for facet width *w* = 32 nm, gap size *d* = 2.4 nm, and refractive index *n*_g_ = 1.56. (e) Absorption (blue) and emission (red) spectra of β-carotene in 25 μM ethanol solution under λ_p_ = 447 nm excitation. Inset shows chemical structure of β-carotene, backbone conjugated over 9 double bonds, with calculated dark-transition Herzberg–Teller dipole (blue) and Franck–Condon quadrupole (orange) orientations. (f) Simplified Jablonski diagram for β-carotene. Bright transition from ground state S_0_ to excited state S_2_ (blue, see calculated Franck–Condon dipole moment) is followed by fast vibrational relaxation (gray) to S_1_, yielding weak PL (red). Note that the fluorescence from S_2_ is also observed. Direct S_0_ → S_1_ photoexcitation is symmetry-forbidden (orange). Calculated vibrationally enabled Herzberg–Teller dipole (**p**) and electronic Franck–Condon quadrupole (**Q**) moments for this transition as indicated.

We exploit plasmonic nanocavities based on a NPoM geometry,^9^ where a monolayer of β-carotene molecules are sandwiched between a gold nanoparticle (diameter *D* = 80 nm) and a gold mirror (Figure 1a). Coulombic coupling between the nanoparticle and its image charges in the mirror gives optical confinement volumes *V* ≃ *Dd*^2^/2*n*_g_^2^ ∼ 100 nm^3^ (for gap size *d* = 2.4 nm and refractive index *n*_g_ = 1.56), providing routine access to field enhancements |***E***|/|***E***_0_| > 100. As the Au nanoparticles are inherently faceted,^33,34^ the NPoM cavity is laterally bounded by the downward-facing nanoparticle facet.^24,35,36^ A set of plasmonic modes are supported in such metal–insulator–metal cavities, out of which the three most radiative ones (labeled by *lm* = 10,11,20) dominate its optical response.^23,24^ Their simulated near-field profiles (Figure 1b) show that higher-order cavity modes, such as (11) and (20), produce substantial field gradients across the *x*–*y* plane of the gap. These gradients (|∇***E***|/|***E***_0_|) are 3000 times larger than those achieved by focusing a laser field with a lens of NA = 1, making them ideally suited for facilitating light-forbidden multipolar transitions.^29^

The resonant wavelengths of the bright nanocavity modes are experimentally resolved by angularly separating the high-angle (θ_high_) and low-angle (θ_low_) dark-field scattering, for each nanostructure (Figure 1c).^37^ Vertical emission (along *z*-direction) from the (11) mode is captured in low-angle scattering spectra, while (10) and (20) preferentially out-couple at high angles (θ ∼ 60°, defined from the vertical direction), spectrally distinguished from the low-angle component.^23^ The experimental mode wavelengths agree with mode positions extracted from finite element method simulations performed with typical parameters for β-carotene nanogaps: *D* = 80 nm, *w* = 32 nm, *n*_g_ = 1.56, and *d* = 2.4 nm (Figure 1d), where the facet width *w* has a linear dependence on the particle diameter (*w*/*D* = 41%)^35^ and *n*_g_ is defined by β-carotene molecules.^38^ Employing the plasmonic spectral resonances in NPoM as an effective plasmonic ruler, an average gap size of 2.4 ± 0.2 nm is estimated by comparing the simulated (10) mode position with the experimental dark-field distribution (Figure S1).^39,40^ With these parameters, we achieve in our simulations a successful alignment of the spectral positions of all three optical modes (Figure 1c,d).^23^

β-carotene is ubiquitous and crucial in photosynthesis^41^ and nutrition.^42^ Strong one-photon absorption from the ground state S_0_ to the singlet state S_2_ in the blue region of the spectrum determines its orange color pigment (Figure 1e,f). However, carotenoids have atypical photophysical organization since the lowest energy excited state S_1_ is one-photon (symmetry) forbidden with respect to the ground state and is located below the dipole-allowed S_2_ state (Figure 1f).^43−45^ The lifetime and energy alignment of the excited states of carotenoids depend on the number of conjugation bonds in the backbone.^41−43^ When the S_2_ state (1^1^B_u_^+^) of all-trans-β-carotene is photoexcited, it internally relaxes into the S_1_ state within ∼200 fs,^46^ which in turn decays to the ground state within several picoseconds.^47,48^ Upon excitation of the S_2_ state, fluorescence from both the dark S_1_ and bright S_2_ states has been observed with very low radiative quantum yields (∼10^–6^ and ∼10^–4^, respectively).^49−53^ In accordance with previous reports, we attribute the broad PL band in solution at ∼700 nm (Figure 1e) to fluorescence from the S_1_ state, and the emission shoulder below 600 nm to S_2_ → S_0_ relaxation. This assignment is also supported by the PL spectrum of β-carotene powder where either S_1_ or S_2_ emission is observed depending on the excitation wavelength (Figure S2). Such residual dark S_1_ state fluorescence originates from the breakdown of the Franck–Condon approximation which neglects the nuclear dependence of transition dipole moments.^30^ Specifically, symmetry-forbidden transitions, such as the S_1_ state of β-carotene may be brightened via vibrations that break the molecular symmetry. Such vibrationally enabled transition moments are commonly referred to as the Herzberg–Teller contribution, which represents a higher-order interaction term going beyond the Franck–Condon approximation, see the Supporting Information.^31,32^

As discussed in more detail below (see also the Supporting Information), quantum chemistry calculations reveal that the transition from the ground state S_0_ (1^1^A_g_^–^) to the lowest excited state S_1_ (2^1^A_g_^–^) presents a Franck–Condon quadrupole moment, |**Q**| = *Q* ≈ 0.06 *e*·nm^2^, and a small vibrationally enabled Herzberg–Teller dipole moment, |**p**| = *p* ≈ 0.006 *e*·nm, both aligned along the molecular backbone (Figures S8–S9). Based on Fermi’s golden rule, the light–matter interaction Hamiltonian for the S_0_ → S_1_ transition can therefore be written as a sum of dipolar and quadrupolar terms of the form
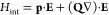
1

**Figure 2:**
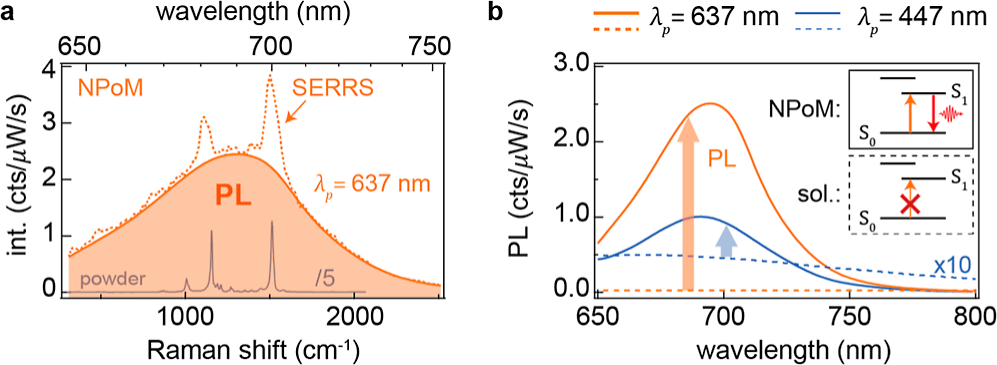
NPoM-modified emission from β-carotene. (a) Surface-enhanced resonance Raman spectrum on PL background from β-carotene in NPoM cavity with λ_*p*_ = 637 nm (orange). Raman spectrum of β-carotene powder is also shown (gray). (b) PL spectrum from β-carotene in NPoM cavity using S_0_ → S_1_ (λ_p_ = 637 nm, solid orange) or S_0_ → S_2_ (λ_p_2__ = 447 nm, solid blue) excitation. PL from β-carotene in solution for the same excitation wavelengths shown for comparison (dashed lines). Inset depicts scheme of excitation and emission in NPoMs and solution. Arrows show enhancement.

As a model molecule, all-trans-β-carotene is thus suitable to investigate the multipolar character of dark transitions, which are expected to be significantly enhanced by large field gradients supported in plasmonic nanocavities. We can estimate the relative weight of the two terms in eq 1 for the PL of the S_1_ → S_0_ transition of β-carotene in free space. Taking the ratio of the emission rates from both terms, we obtain (details in the Supporting Information)
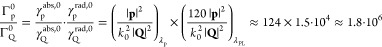
2where *k*_0_ = 2π/λ is the wave-vector in free space. The first term in the products of eq 2 accounts for the ratio between absorption (or in-coupling) efficiencies and is evaluated at the experimental pumping wavelength (see below, λ_p_ = 637 nm). The second term, which corresponds to the radiative (or out-coupling) rates,^26^ is calculated at the maximum PL as shown in Figure 2b (λ_PL_ = 700 nm). We conclude that despite its small value, the emission in free space from this dark molecular transition is fully governed by its Herzberg–Teller dipole moment. In the following, we explore if this is also the case when the β-carotene molecules are placed within the gap of NPoM nanocavities.

Intriguingly, despite the absence of optically allowed excited states below S_2_, lower energy excitation (λ_*p*_ = 637 nm) of β-carotene molecules within a majority (90%) of randomly selected NPoM cavities results in pronounced PL from the S_1_ state, centered around 700 nm (Figure 2a, solid orange; Figure S3). This is accompanied by additional surface-enhanced resonance Raman scattering (SERRS) (Figure 2a, dashed line) matching the Raman peaks obtained from β-carotene bulk powder (Figure 2a, gray line). Direct excitation of the S_2_ state at 447 nm also yields the same S_1_ state emission in NPoMs (Figure 2b, solid blue line), although its total PL quantum efficiency is 5-fold lower compared to 637 nm excitation. Such emission is completely absent from NPoM cavities without β-carotene molecules, which have red-shifted dark-field resonances (due to the smaller gap size) and lack any SERS response.

The NPoM-modified emission as shown in Figure 2 is in strong contrast to the results for β-carotene powder. In the case of β-carotene powder when directly exciting the S_2_ state, the PL quantum efficiency is found to be 260 times higher compared to the excitation of the S_1_ state (Figure S2). The experimental PL enhancement factor (EF_e_) per molecule of β-carotene in NPoMs with respect to in ethanol solution, is estimated using
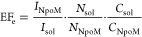
3where, with 637 nm excitation of the S_1_ state, *I*_NPoM_ = 190 cts/μW/s and *I*_sol_ = 1.0 cts/μW/s are the integrated experimental PL intensity in NPoM cavities and in ethanol solution, respectively, *N*_NPoM_ ≈ 50 and *N*_sol_ ≈ 3 × 10^4^ are the numbers of molecules excited in each case (see the Supporting Information), and *C*_NPoM_ = 0.33 and *C*_sol_ = 0.29 are the collection efficiencies in the two different measurement schemes with a 0.8 NA objective.^14^ Using these parameters, EF_e_ ∼ 1.3 × 10^5^ is obtained for NPoMs compared to solution.

The observed enhancement factors of β-carotene within the NPoM system are significantly high, surpassing the EF_e_ achieved using low quantum yield emitters by 2 orders of magnitude.^6,13^ This indicates that dipole in-/out-coupling enhancement is inadequate to account for the emission efficiency observed experimentally. Consequently, a detailed investigation into the role of quadrupole enhancement in PL from NPoM cavities at low energy excitation is needed. Contrary to the negligible impact of the quadrupole interaction in free space (as delineated in eq 2), within the NPoM system, the quadrupole term in eq 1 assumes a substantial role. For comparison, when directly exciting the S_2_ state at λ_p_2__ = 447 nm, EF_e_ = 100 is estimated for the β-carotene PL. This enhancement is mostly attributed to the cavity-boosted out-coupling efficiency at 690 nm, given the relatively weak field confinement at the 447 nm excitation wavelength.

We exploit the fact that the out-coupling rate is independent of the excitation wavelength to further investigate the quadrupole contribution to the optical absorption of β-carotene molecules through the S_0_ → S_1_ transition. First, we evaluated the dipole-assisted absorption at both pumping wavelengths. Quantum chemistry calculations indicate that the transition dipole moment for S_1_, p, is roughly 80 times lower than that for S_2_, p_2_. Electromagnetic simulations (see maxima in the dipole-absorption spectrum, |***E***_∥_/***E***_0_|^2^, in Figure S10) reveal that the maximum field enhancement in the NPoM cavity is  times higher at λ_p_ = 637 nm than at λ_p_2__ = 447 nm (|***E***_∥_/E_0_|_447 nm_^2^ ≈ 1, as the cavity does not sustain bright plasmonic modes in this spectral range), thus resulting in a ratio between absorption rates, . Therefore, even in the optimum scenario, the enhancement in dipolar absorption at the dark transition through the NPoM plasmonic modes is not sufficient to explain the contrast in the experimental PL efficiencies observed in Figure 2b (∼5× higher under 637 nm excitation). This result provides further evidence that there is an additional quadrupolar contribution to the optical absorption of the S_0_ → S_1_ transition.

**Figure 3:**
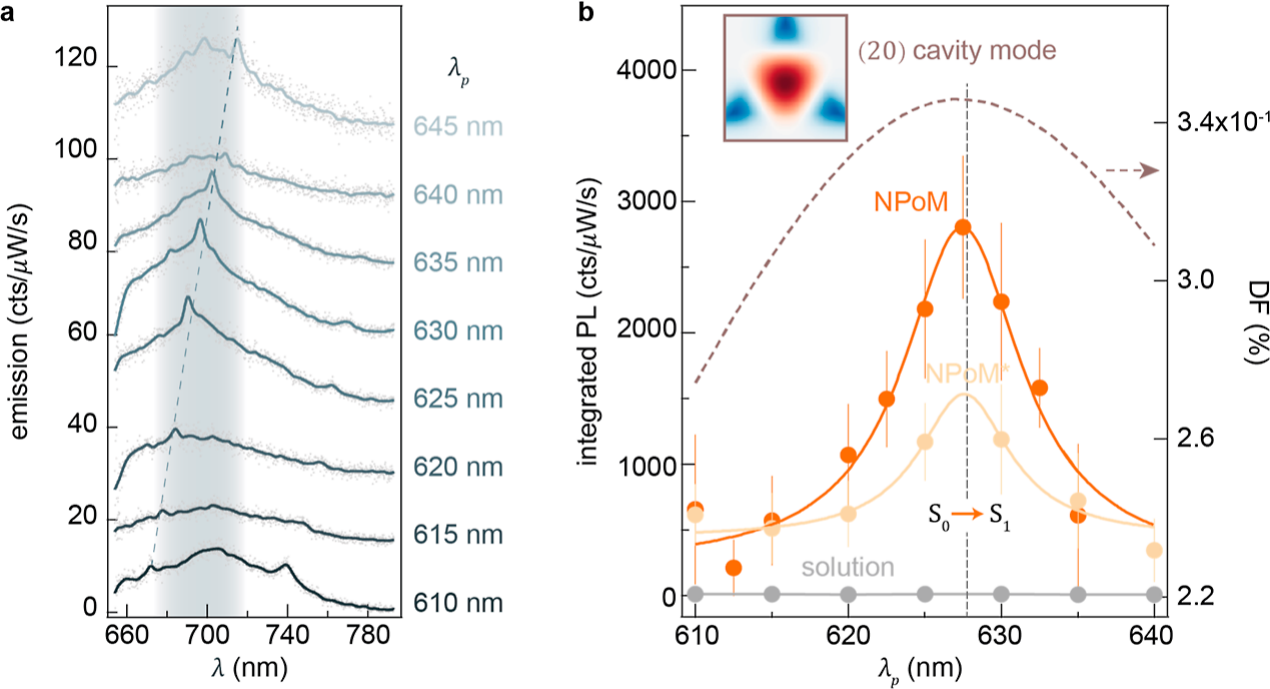
S_0_→ S_1_ absorption enhancement. (a) Emission spectra for a single β-carotene-filled NPoM vs pump excitation tuned from 610 to 645 nm, vertically offset for clarity. Dashed lines mark molecular Raman resonances at 1520 cm^–1^ (C=C stretch). (b) PL excitation spectra of β-carotene (orange)- and β-carotene⊆cucurbit[7]uril (yellow)-filled NPoMs, compared to β-carotene in solution (gray). Solid curves are Lorentzian fits; error bars give standard deviation from 45 NPoM cavities. Dark-field scattering resonance of (20) cavity mode (dashed curve) with (inset) its near-field map under a triangular facet.

To manifest the wavelength dependence of the absorption enhancement, we collect the S_1_ → S_0_ emission spectra while scanning the excitation wavelength (λ_p_) from 610 to 645 nm for 45 individual β-carotene NPoMs (Figure 3a). Using 1 ps excitation pulses reduces the laser line width enough to be able to trace both the SERRS lines and the broadband PL as λ_p_ is tuned. The PL spectra yield a consistent emission band from the S_1_ state centered at around 700 nm. This emission is independent of λ_p_ and is identical for different NPoMs, confirming the molecular origin of the emission. By comparison, the molecular SERS peaks from the C=C stretch at 1520 cm^–1^ and the CH stretch at 2870 cm^–1^, shift in wavelength according to λ_p_. A SERRS resonance is observed as the C=C Raman line tunes across the S_1_ emission wavelength, being enhanced along with the PL intensity and maximized where the Raman resonance and S_1_ peak overlap (Figure S4). Since phonon-assisted absorption modifies selection rules, we note that phonon-assisted relaxation might also play a role in the NPoM-enhanced molecular emission.^48^

The fitted PL excitation spectra show that the integrated S_1_ emission peaks at λ_p_ = 627 ± 1 nm for NPoM cavities filled with β-carotene molecules (Figure 3b, orange line). We can also encapsulate each β-carotene in a cucurbit[7]uril (CB[7]) molecule to better separate and scaffold them,^54^ but the resonant behavior remains very similar (labeled NPoM* in yellow). Beneficially, this also matches the measured scattering resonance of the (20) NPoM cavity mode (Figure 3b, dashed line). As discussed above, this higher-order plasmonic cavity mode exhibits desirable radiative efficiencies while supporting significant optical field gradients capable of enhancing quadrupole transitions.^23,29^ As a control measurement, the PL excitation spectrum of β-carotene solution is uniformly negligible across this excitation wavelength range (Figure 3b, gray), concurring with the negligible dipolar absorption in optical extinction (Figure 1e).

To quantitatively assess the weight of Herzberg–Teller dipole and Franck–Condon quadrupole contributions to the absorption and radiative efficiency of the S_0_–S_1_ transition in the NPoM cavities, we perform electromagnetic simulations for single molecules placed at different positions within the gap facet area. The enhancement factors for purely dipolar (**p**) and quadrupolar (**Q**) transitions are defined as  and .^55^ The radiative Purcell enhancement of the molecular dipole within the NPoM cavity is determined by
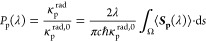
where κ_p_^rad,0^ (λ) = 8π^2^ |**p**|^2^/3λ^3^ ε_0_ ℏ, ⟨*S*p (λ)⟩ represents the numerically obtained time-averaged Poynting vector from the dipolar source **p** placed in the NPoM gap and Ω denotes the far-field surface that accounts for the dark-field collection angle (with NA ≈ 1 to reflect the experimental high-angle collection). For the quadrupolar case, the enhancement is given by
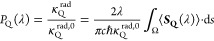
with κ_p_^rad,0^ (λ) = 4π^4^ |**Q**|^2^/45λ^5^ ε_0_ ℏ and ⟨*S*_Q_ (λ)⟩ as the time-averaged Poynting vector from the quadrupolar source **Q** in the NPoM gap. Our estimation of the gap size (*d* = 2.4 ± 0.2 nm) from the scattering spectra suggests that the β-carotene molecules (∼3 nm long, from quantum chemistry calculations) are not vertically aligned to the facets in the experimental samples. For this reason, we analyze in detail the influence of tilting angle, θ, on the PL.

**Figure 4:**
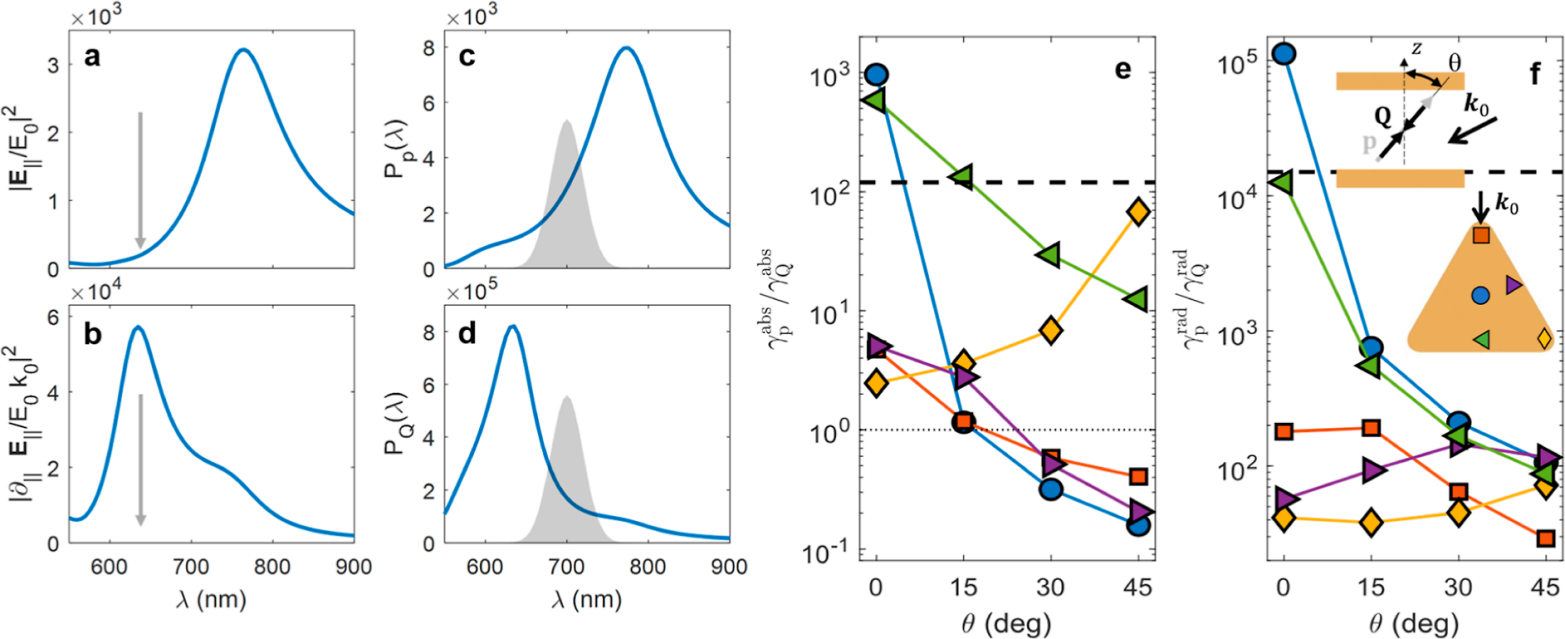
Theoretical dipole and quadrupole contributions to S_1_ → S_0_ PL emission. (a,b) Dipole and quadrupole absorption enhancement spectra for a β-carotene molecule at the facet center, tilted 30° with respect to the vertical direction (vertical arrow marks λ_p_). (c,d) Dipole and quadrupole radiative Purcell spectra for the same position and orientation (the shaded area represents the molecule PL spectrum). (e) Ratio between Herzberg–Teller dipole (**p**) and Franck–Condon quadrupole (**Q**) optical in-coupling rates as a function of the molecular orientation for β-carotenes at different locations under the NP facet (colors). (f) Same as (e) but for the out-coupling rates. The insets sketch the molecule position and orientation within the incident plane and relative to the direction of incidence, ***k***_0_.

Figure 4a–d shows the in-coupling (left) and out-coupling (right) enhancement spectra (normalized to free space) for a dipolar (top) and a quadrupolar (bottom) transition for a molecule at the facet center and θ = 30°. As anticipated, the dipole spectra are governed by the (10) NPoM plasmonic mode, while the (20) mode governs the quadrupole ones. The vertical gray arrows in Figure 4a,b mark the experimental pumping wavelength, which is resonant with the latter mode. This fact, together with the large field gradients associated with the resonant electric field yield absorption rate enhancements ∼100 times larger for the quadrupole moment. Figure 4c,d shows the wavelength-dependent radiative Purcell factors along with the free-space PL spectrum (gray shaded area), which is significantly detuned from both nanocavity modes. The extremely large local density of photonic states supported by the (20) mode gives rise to another ∼100-fold larger out-coupling enhancement for the quadrupole transition. Thus, by simple inspection of Figure 4a–d, and using eq 2, we can conclude that the S_1_ → S_0_ emission at this particular position and orientation is still governed by the Herzberg–Teller dipole, since  ≈ 180. This ratio indicates that the FC quadrupole is no longer negligible, in contrast to the situation in free space where this ratio is ∼10^6^. It is crucial to recognize, however, that this estimation unphysically assumes that the fluorescence mechanisms mediated by the Herzberg–Teller dipole and Franck–Condon quadrupole moments are entirely independent. Realistically a strong interplay can occur between these mechanisms. As we discuss further below, our theoretical analysis reveals that, despite its minimal radiative contribution, the quadrupolar character of the S_0_ → S_1_ transition significantly influences the β-carotene absorption within the NPoM cavity.

Figure 4e presents a comprehensive study of the ratio between in-coupling rates of the Herzberg–Teller dipole and Franck–Condon quadrupole for different molecule positions (see the inset of Figure 4f) and orientations (see the Supporting Information for further details on the calculation procedure). The ratio in free space and the condition γ_p_^abs^/γ_Q_^abs^ = 1 are marked by horizontal black dashed and dotted lines, respectively. We can observe that for vertically oriented molecules located at the center under the NP facet the disparity between quadrupole and dipole absorption is more pronounced. However, for most configurations, this difference is substantially diminished, so quadrupole contributions are more important away from the facet center. Intriguingly, at high tilt angles and at edge and corner positions directly exposed to the incident fields, the Franck–Condon quadrupole absorption rate surpasses its Herzberg–Teller dipole counterpart by up to a factor of 10, likely due to the result of edge effects. This observation underscores the dominant quadrupolar nature of the absorption of the dark β-carotene transition in these experimentally relevant cases. This is in good agreement with our experimental data, suggesting that a purely dipolar enhancement cannot adequately account for the observed PL enhancement in the NPoM samples.

Finally, we investigate the dipole-to-quadrupole radiative rate ratio, as depicted in Figure 4f. The NPoM dramatically reduces the discrepancy between the two out-coupling mechanisms, resulting in a lower ratio compared to free space (horizontal dotted line) for all considered configurations, except for central, vertically oriented molecules. Nevertheless, γ_p_^rad^/γ_Q_^rad^ > 10 in all cases, indicating that, contrary to its absorption characteristics, the molecular radiation in our NPoM-β-carotene samples predominantly exhibits a Herzberg–Teller dipolar nature. Notably, however, this dominance is considerably reduced in tilted molecules under direct illumination.

The intricate complexity of such β-carotene NPoM samples suggests the potential involvement of a variety of distinct mechanisms that may contribute to the observed PL. These mechanisms range from cavity-induced molecular charge transfer or collective interactions to the initiation of photochemical reactions within the nanogap. The data above constrain these (for instance, since the dark field resonances do not shift with irradiation). Additionally, the substantial electric field gradients within the plasmonic cavity can generate AC magnetic fields, suggesting that magnetic dipole mechanisms could also play a role in modulating the transition rates. Our combined experimental and theoretical analysis presented above thus elucidates the influence exerted by the quadrupolar character of the dark S_0_ → S_1_ transition on the PL of β-carotene NPoM systems. Further experimental confirmation would require direct tuning of the optical field gradients (without changing the resonance conditions), perhaps using mechanically translated nanoparticles using tip-based scanning microscopies.

## Conclusions

In this study, we elucidated the far-field probing of optically forbidden molecular transitions using tightly confined plasmonic fields supported by metallic nanocavities. In particular, we experimentally examined the PL from an elusive S_1_ → S_0_ transition of β-carotene molecules positioned within the gap of NPoM plasmonic cavities. We observe an emission enhancement from our samples that surpasses the limitations of explanations solely relying on dipolar channels. Through examination of the PL excitation spectrum, we identify a notable alignment with the resonance of a higher-order plasmonic cavity mode where strong field gradients are supported. Through a numerical analysis combining quantum chemistry and electromagnetic calculations, we have found that the Franck–Condon quadrupolar contribution to optical absorption by the molecules, negligible in free space, becomes comparable to, and even larger than, the vibrationally enabled Herzberg–Teller dipolar mechanism, proving the multipolar character of the β-carotene light-forbidden transition. Our results facilitate the exploitation of optically inactive material excitations in photon-based techniques ranging from spectroscopy or sensing to imaging or microscopy.

## Materials and Methods

### Sample Preparation

To form a SAM, all-trans-β-carotene (Sigma-Aldrich) is dissolved in 25 μM ethanol solution with 10 min sonication at room temperature. Template-stripped gold substrates are immersed in β-carotene solution for 24 h to allow the SAM to saturate. The samples are rinsed with pure ethanol before being blow-dried with nitrogen. For β-carotene⊆cucurbit[7]uril samples, β-carotene and cucurbit[7]uril (Sigma-Aldrich) are mixed in a 1:1 molar ratio in ethanol. To create the NPoM geometry, 80 μL of AuNP dispersion (BBI Solutions, *D* = 80 nm) is drop-cast onto the β-carotene-coated gold substrate. Excess AuNPs are washed off with deionized water after 40 s of deposition, followed by drying with nitrogen.

### Optical Measurement

PL measurement is performed with a customized dark field microscope (Olympus BX51) in reflection geometry. A CW diode laser at 447 or 637 nm (Coherent CUBE) is spectrally filtered before coupling to the microscope through a single mode fiber. The excitation laser is collimated to fill the back focal plane of a 100× Olympus objective (0.8 NA). Individual NPoM constructs are excited with a diffraction limited illumination spot while solution samples are measured in quartz cuvettes. The reflected laser light is blocked by long-pass filters (Edmund Optics) in the collection path where PL signals are captured with a cooled spectrometer (Ocean Optics QE65000).

PL excitation spectroscopy is realized using the experimental setup detailed in ref (14). Spectrally tunable pulses (500–720 nm) from an 80 MHz pumped Spectra-Physics optical parametric oscillator are spectrally filtered to yield a 1 ps pulse duration. The optical power of the pulses is controlled by using an automated continuously variable neutral density filter. The attenuated pulses are coupled to the microscope through free space and focused by a microscope objective NA = 0.9. Emission signals are spectrally filtered by using long-pass interference filters and recorded on a grating spectrometer system (Andor EMCCD).

Energy momentum spectroscopy is performed using a modified Olympus BX51 microscope in reflective dark field geometry. Individual NPoMs are illuminated with focused incoherent halogen source at an annular illumination angle of 64–75° with respect to normal incidence, and scattered light of <64° is collected through a dark-field objective (Olympus, NA 0.9). Single NPoM structures are spatially isolated by spatially filtering the real image plane with a pinhole. The scattering pattern from a NPoM at the back focal plane of the microscope objective is demagnified by 3 times before being imaged on the entrance slit (150 μm wide) of a Triax 320 spectrometer, where a narrow range of the scattering pattern near *k*_*x*_/*k*_0_ = 0 is filtered and dispersed by a 150 L/mm grating and recorded using an Andor Newton 970 BVF EMCCD.

### Quantum Chemistry Calculations

Electronic structure calculations with the Q-Chem and Gaussian software packages were carried out to estimate the relative magnitudes of the transition Franck–Condon quadrupole and Herzberg–Teller dipole moments of the dark S_1_ excited state of β-carotene. All the details of the calculations can be found in the Supporting Information.

### Electromagnetic Simulations

Finite element simulations were performed using the frequency-domain solver of Maxwell’s equations implemented in commercial software Comsol Multiphysics. NPoM cavity geometry and material characteristics were set using the nominal parameters extracted from the experimental samples. Absorption spectra were obtained by solving the scattering problem under grazing plane-wave illumination. Point-like dipolar and quadrupolar currents were used as the electromagnetic source in the calculation of the radiative Purcell spectra. Details of the procedure employed in the calculation of in/out-coupling and PL rates can be found in the Supporting Information.

## Supporting Information Available

Histogram of β-carotene NPoM cavity resonances; PL spectra of β-carotene powder; additional statistical analysis of NPoM PL; PL and surface-enhanced Raman scattering excitation resonance; angle-resolved emission from β-carotene NPoM; emission spectra of β-carotene NPoMs with varied pump wavelength; analysis of quantity of molecules under optical excitation; computational details and discussion on electronic structure calculations; and electromagnetic simulations on absorption and Purcell factor (PDF)
