# Supplementary material for: Influence of Quadrupolar Molecular Transitions within Plasmonic Cavities
Source: ACS Nano. 2024 May 24;18(22):14487–95. doi: 10.1021/acsnano.4c01368 (PMC11155255; doi:10.1021/acsnano.4c01368)
Supplement: Supplementary file 1 — nn4c01368_si_001.pdf [file nn4c01368_si_001.pdf]

Supplementary Information for

The Influence of Quadrupolar Molecular Transitions within Plasmonic Cavity Modes

Junyang Huang[1], Oluwafemi S. Ojambati[1,†], Clàudia Climent[2,3], Alvaro Cuartero-Gonzalez[2,4], Eoin Elliott[1], Johannes Feist[2], Antonio I. Fernández-Domínguez[2*], Jeremy J. Baumberg[1*]

[1]NanoPhotonics Centre, Cavendish Laboratory, Department of Physics, JJ Thompson Avenue, University of Cambridge, Cambridge, CB3 0HE, UK

[2]Departamento de Física Teórica de la Materia Condensada and Condensed Matter Physics Center (IFIMAC), Universidad Autónoma de Madrid, E-28049 Madrid, Spain

[3]Department of Chemistry, University of Pennsylvania, Philadelphia, Pennsylvania 19104, USA

[4]Mechanical Engineering Department, ICAI, Universidad Pontificia Comillas, 28015 Madrid, Spain

[*]Corresponding authors. Email: jjb12@cam.ac.uk (J.J.B.), a.fernandez-dominguez@uam.es (A.I.F.-D.)

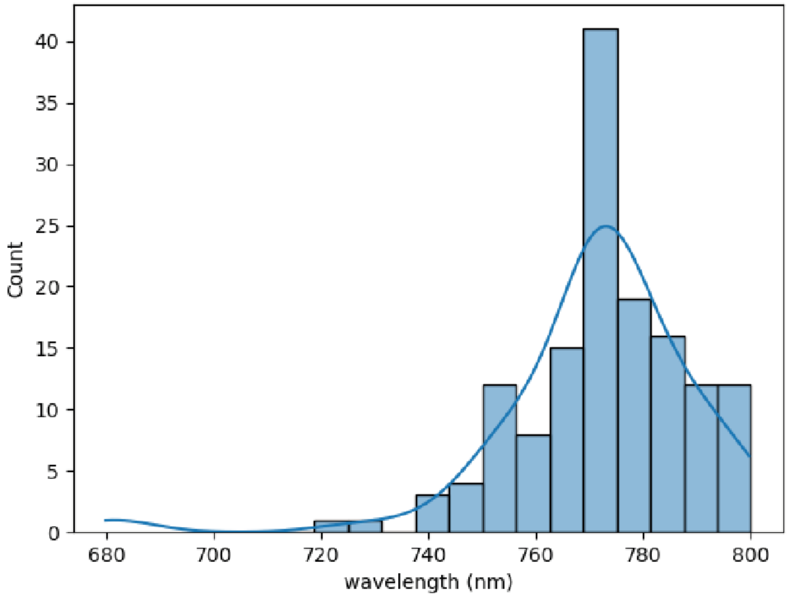

Fig. S1. Distribution of (10) cavity mode resonances in dark-field scattering for >130 β-carotene NPoMs (nominal D_{AuNP} = 80 nm).

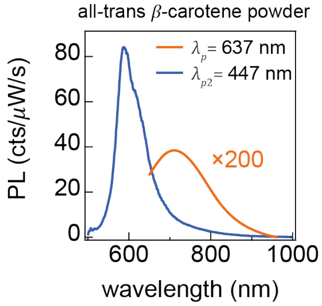

Fig. S2. Photoluminescence (PL) from *all-trans-β*-carotene powder with λ_p = 637 nm (orange) and λ_{p2} = 447 nm (blue). PL intensity with λ_p = 637 nm is scaled by 200 times to help visualize the $S_1 \rightarrow S_0$ emission.

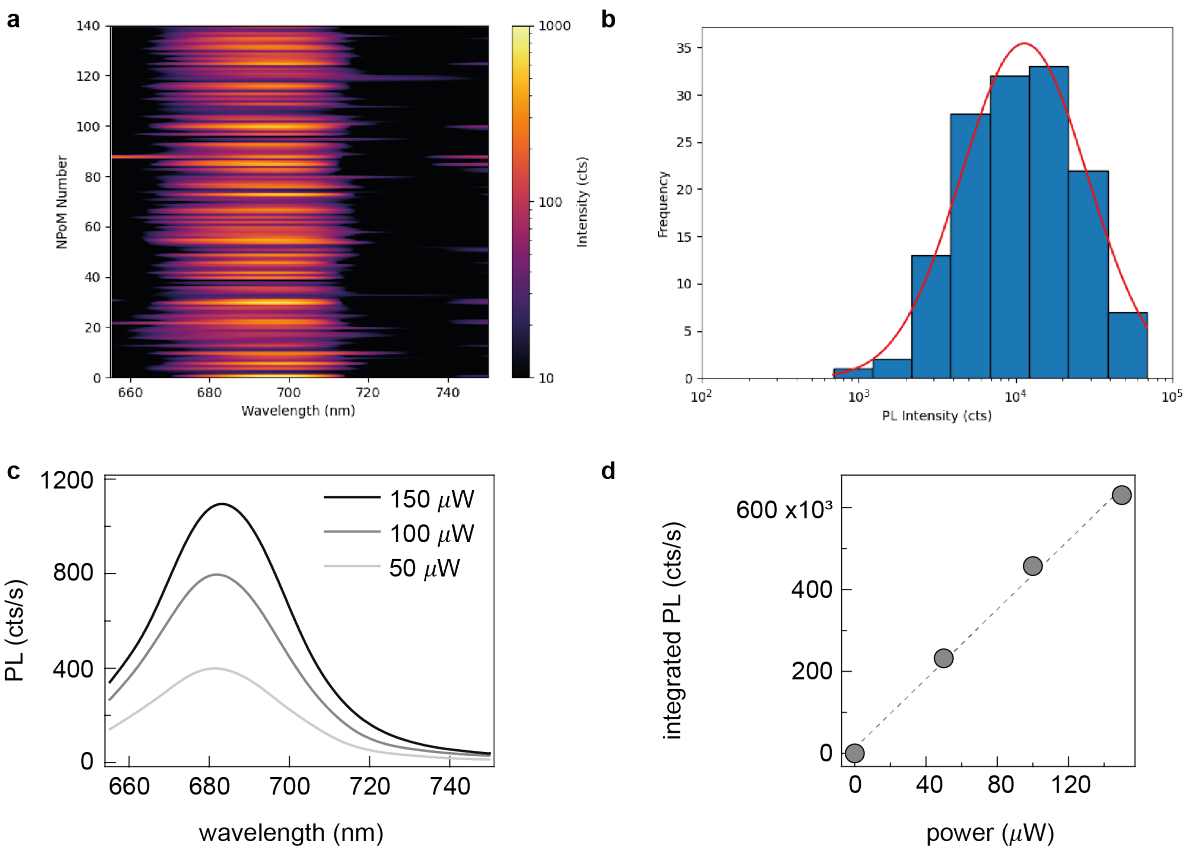

Fig. S3. (a) PL spectra from 140 beta-carotene NPoMs under 633 nm CW excitation, showing consistent emission from the S_1 state. (b) Histogram of total integrated PL intensities, with a Gaussian fit (red). (c) PL spectra for a β-carotene NPoM with increasing CW excitation power (λ_p=633nm). (d) Power dependence of emission showing linearity with incident laser power.

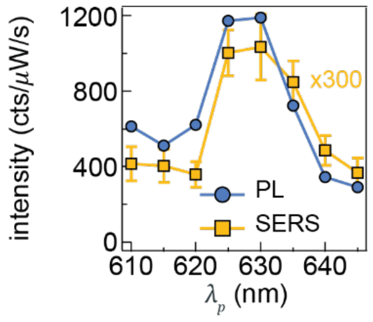

Fig. S4. PL (blue) and SERS intensity of the 1520 cm^{-1} line (yellow) from β-carotene NPoM with excitation wavelengths from 610 to 645 nm. SERS intensity is scaled by 300 times in the plot.

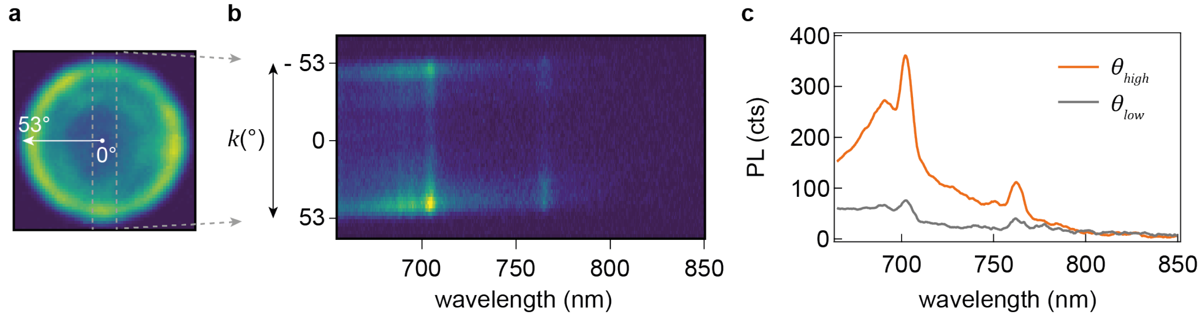

Fig. S5. Angle-resolved emission from beta-carotene NPoM. (a) Back focal plane (BFP) photoluminescence image captured from single β-carotene NPoM cavity with NA=0.8 dark field objective, showing high angles dominate emission (spectrally integrated). (b) Energy-momentum spectrum captured by filtering wavevector range (dashed line in a) of the BFP image near $k_x/k_0=0$. (c) Angle-resolved emission spectra of β-carotene NPoMs.

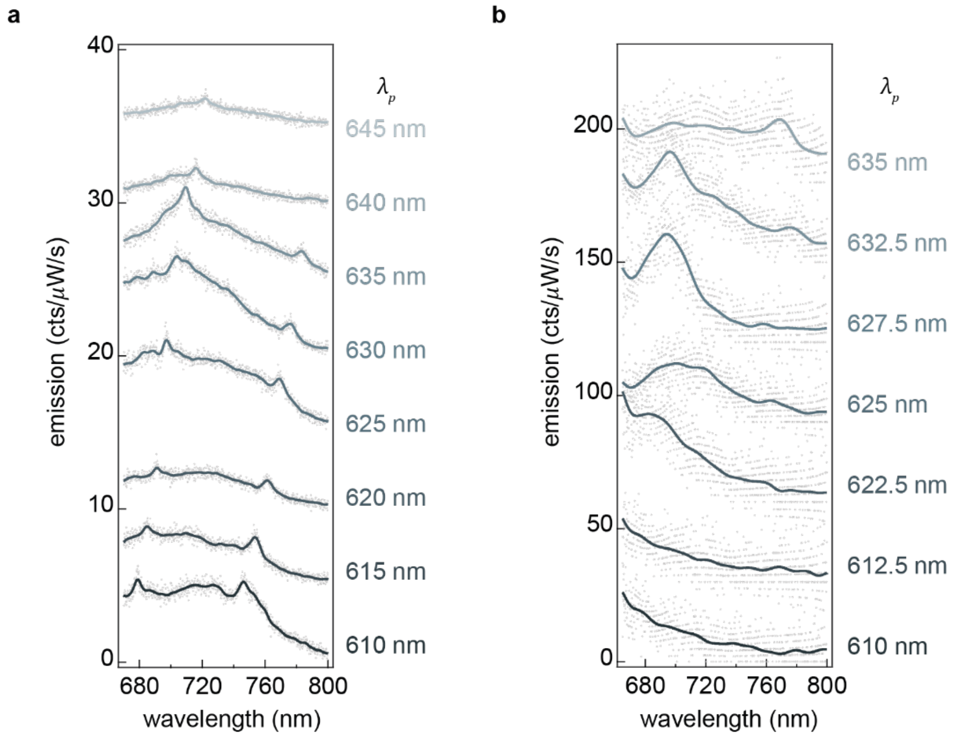

Fig. S6. (a) Median emission spectra of 45 β-carotene NPoMs with increasing pump wavelength from 610 to 645 nm. (b) Emission spectra of a single β-carotene⊆CB[7] NPoM with increasing pump wavelength. Spectra offset for clarity.

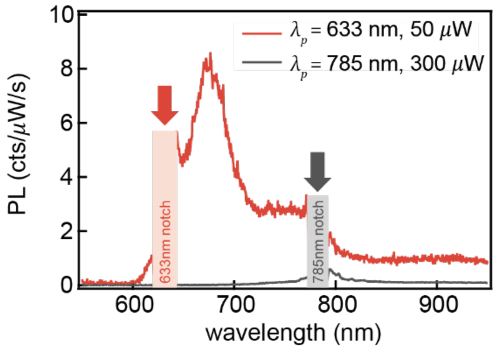

Fig. S7. Emission spectra of β-carotene from NPoM cavity with 633 nm (red) and 785 nm (grey) CW excitation.

To exclude the possibility that the 680 nm emission observed under $S_0 \rightarrow S_1$ excitation does not originate from Raman scattering, we directly compare the NPoM emission using 633 nm and 785 nm laser excitation. The absence of emission under 785 nm excitation corroborates that the $S_0 \rightarrow S_1$ excited emission observed around 680 nm is not due to Raman scattering. Note that these spectra are recored using a Triax 320 spectrometer and an Andor Newton 970 BVF EMCCD, therefore exhibit different spectral resolution and sensitivity to that shown in Figure 2.

Quantity of molecules under optical excitation

The area of a triangular facet is 443 nm^2 for an edge width w=32 nm. The separation between the nearest neighbour head groups was measured to be 3 nm in previous STM studies.[1] Assuming the most compact hexagonal packing for the molecules in a self-assembled monolayer, we estimate that each molecule occupies an area of 8 nm². Consequently, we establish an upper bound for the number of molecules under the facet by dividing the facet area by the area occupied by each molecule, yielding $N_{\mathrm{NPoM}} \approx 50$.

To determine N_{sol} in a 25 μM β-carotene solution irradiated by a focused laser beam with a spot size w_0 of 0.5 µm (wavelength λ = 633 nm, numerical aperature = 0.8), and Rayleigh length $z_R$of 1.2 μm ($Z_R = \pi w_0^2/\lambda$), the volume of the focused laser spot is approximated to be $v_0 = 2\pi w_0^2 z_R \approx 1.9 \times 10^{-18}$ m^3. Within this focal volume, the estimated number of molecules present in the 25 μM solution is $N_{\mathrm{sol}} \approx 3 \times 10^4$.

Electronic structure calculations: computational details and discussion

To estimate the order of magnitude of the electronic transition quadrupole moment of the $^1A_g^-$ (S_1) excited state of all-trans beta-carotene, time-dependent density functional theory calculations were carried out with and without the Tam–Dancoff approximation (TDA and TDDFT) with an in-house developer's version of the Q-Chem 5.4 package.[2] The ground state geometry

was optimized at the B3LYP/6-31G(d) level, while the CAM-B3LYP and ωB97X-D exchange-correlation functionals as well as the aug-cc-pVDZ basis set were used for excited state calculations.[3,4] The (non-traceless) transition quadrupole moment tensor was calculated in the length gauge with the origin placed at the center of nuclear charge.

In Table S1 we report the calculated electronic transition dipole and quadrupole moments between the ground state and the dipole-allowed, $^1B_u^+$ (S_2), and forbidden, $^1A_g^-$ (S_1), excited states. The $^1B_u^+$ state mainly corresponds to a HOMO→LUMO transition while the $^1A_g^-$ state is mostly a HOMO-1→LUMO transition with some HOMO→LUMO+1 contribution. We should note that it is well-known that double excitations (HOMO^2→LUMO^2) contribute to the $^1A_g^-$ excited state of long polyenes.[5–7] Unfortunately, TDDFT cannot account for such double excitations, and this is precisely the reason why the relative energies of the $^1A_g^-$ and $^1B_u^+$ are not well-captured: while it is known that $^1A_g^-$ and $^1B_u^+$ correspond to the first and second excited singlet states, respectively, TDDFT (and TDA) usually predicts the reverse order.[8,9] Still, as can be seen from Table S1, the dipole allowed/forbidden nature of these states is well-captured with TDDFT, since it depends on the symmetry of the states which is dictated by the molecular orbital contributions.[6]

Additional comments are due regarding the impact of having double excitations in the transition quadrupole moment (as calculated within TDA where one can think of an excited state as a CIS wavefunction, i.e., linear combination of singly excited Slater determinants). According to the Slater-Condon rules,[10] the matrix element of a one-electron operator between two Slater determinants that differ in (at least) two orbitals (e.g., ground state wavefunction with doubly occupied orbitals and a doubly excited configuration) is zero. The presence of doubly excited contributions in the $^1A_g^-$ excited state would reduce the weight of the singly-excited ones, and thus the transition quadrupole moment with the ground state is expected to be slightly smaller than the values reported in Table S1. This would be the case unless the double excitation also contributes to the ground state wavefunction of all-trans beta-carotene, as has been reported for long polyenes,[7] in which case, there would be a net contribution to the transition quadrupole moment. Note that the values reported in Table S1 were calculated at the optimized ground state geometry. We have verified that the transition quadrupole moment calculated at the $^1A_g^-$ excited state equilibrium geometry is of the same order of magnitude.

basis set	method	functional	p (e·nm)		Q (e·nm^2)	
			$^1B_u^+$ (S2)	$^1A_g^-$ (S1)	$^1B_u^+$ (S2)	$^1A_g^-$ (S1)
6-31G(d)	TDDFT	B3LYP	0.44	$5{\cdot}10^{-8}$	$4{\cdot}10^{-8}$	0.08
		CAM-B3LYP	0.42	$2{\cdot}10^{-7}$	$6{\cdot}10^{-8}$	0.32
		ωB97XD	0.42	$2{\cdot}10^{-7}$	$6{\cdot}10^{-8}$	0.40
	TDA	B3LYP	0.50	$5{\cdot}10^{-8}$	$5{\cdot}10^{-8}$	0.07
		CAM-B3LYP	0.46	$9{\cdot}10^{-8}$	$5{\cdot}10^{-8}$	0.18
		ωB97XD	0.45	$2{\cdot}10^{-7}$	$5{\cdot}10^{-8}$	0.37
aug-cc-pVDZ	TDDFT	B3LYP	0.45	$5{\cdot}10^{-8}$	$5{\cdot}10^{-8}$	0.09
		CAM-B3LYP	0.43	$2{\cdot}10^{-7}$	$6{\cdot}10^{-8}$	0.34
		ωB97XD	0.43	$2{\cdot}10^{-7}$	$7{\cdot}10^{-8}$	0.41
	TDA	B3LYP	0.51	$5{\cdot}10^{-8}$	$5{\cdot}10^{-8}$	0.07
		CAM-B3LYP	0.46	$9{\cdot}10^{-8}$	$5{\cdot}10^{-8}$	0.23
		ωB97XD	0.45	$2{\cdot}10^{-7}$	$6{\cdot}10^{-8}$	0.41

Table S1. Calculated (Frank-Condon) transition dipole ($p = \sqrt{p_x^2 + p_y^2 + p_z^2}$) and quadrupole ($Q = \sqrt{Q_{xx}^2 + Q_{yy}^2 + Q_{zz}^2 + Q_{xy}^2 + Q_{xz}^2 + Q_{yz}^2}$) moments for the B_u^+ and A_g^- singlet excited states of all-trans beta-carotene at the equilibrium ground state geometry (optimized at the B3LYP/6-31G(d) level).

The transition moments in Table S1 have been calculated within the standard Franck-Condon (FC) approximation, in which the transition moment is assumed to be constant across the width of the ground-state nuclear wave packet, i.e., independent of nuclear geometry. However, for symmetry-forbidden transitions such as the absorption of the first excited singlet state of beta-carotene, where the transition dipole moment is suppressed at the equilibrium geometry for symmetry reasons, this approximation can break down. Nuclear motion can break the molecular symmetry and is responsible for the vibration-assisted Hertzberg-Teller (HT) contribution to the transition dipole moment, which can thus become the dominant term in dipole-forbidden transitions.

Additional electronic structure calculations were carried out to estimate the order of magnitude of the vibrationally-allowed transition dipole moment of the $^1A_g^-$ excited state of all-trans beta-carotene. Within the Born-Oppenheimer and harmonic approximations, the transition dipole moment between the ground Ψ_g and an excited state Ψ_e may be estimated by the following expression, where the dipole moment is expanded by a Taylor series about the equilibrium (eq) geometry of the excited state[11]

$$\langle \Psi_g | \hat{\boldsymbol{p}} | \Psi_e \rangle = \boldsymbol{p}_{el}(\boldsymbol{X}_{eq}) \left\langle \chi_{g,\vec{v}_i} \middle| \chi_{e,\vec{v}_f} \right\rangle + \sum_{m=1}^{N} \left(\frac{\partial \boldsymbol{p}_{el}}{\partial \boldsymbol{X}_m} \right)_{eq} \left\langle \chi_{g,\vec{v}_i} \middle| \boldsymbol{X}_m \middle| \chi_{e,\vec{v}_f} \right\rangle$$

Note that the molecular wavefunctions are taken as products of an electronic and vibrational component, $|\Psi_g\rangle=|\psi_g\rangle|\chi_{g,\vec{v}_i}\rangle$ and $|\Psi_e\rangle=|\psi_e\rangle|\chi_{e,\vec{v}_f}\rangle$, where $\vec{v}$ is a vector indicating the quanta of each vibrational mode. The first term in the above equation corresponds to the Frank-Condon (FC) contribution, where the electronic transition dipole moment $\boldsymbol{p}_{el} = \langle\psi_g|\boldsymbol{p}|\psi_e\rangle$ is routinely obtained from electronic structure calculations, while $\langle\chi_{g,\vec{v}_i}|\chi_{e,\vec{v}_f}\rangle$ is the Frank-Condon overlap between vibrational wavefunctions. The second term corresponds to the Herzberg-Teller (HT) contribution which accounts for the nuclear dependence of the electronic transition dipole moment. The summation runs over the N normal modes of vibration of the excited state, $\boldsymbol{X}_m$. This HT term is relevant for dipole-forbidden transitions, that is, when $\boldsymbol{p}_{el} \approx 0$. Since the low-lying $^1A_g^-$ state of all-trans beta-carotene is dipole-forbidden (Table S1), we calculated the HT contribution to the transition dipole moment as implemented in the Gaussian package.[12] In particular, the mixing between normal modes of the ground and excited states is accounted for via the Duschinsky transformation,[13] and the zero-temperature limit is considered, meaning that all transitions are calculated starting from the vibrational ground state of the initial electronic state (i.e., $\vec{v} = 0$).[14] Note that calculating the HT contribution requires geometry optimization and frequency calculations of both the ground and the excited state of interest.

Additional remarks are necessary here. Our goal is to estimate the relative order of magnitude of the HT transition dipole moment, p, vs the FC transition quadrupole moment, Q, of the $^1A_g^-$ excited state. Note that the values reported in Table S1 do not include the vibrational contribution from the FC overlaps. This is because the Frank-Condon overlaps just distribute the (FC) electronic transition moment among the different vibronic transitions, with $\sum_f \left|\langle\chi_{g,\vec{v}_i}|\chi_{e,\vec{v}_f}\rangle\right|^2$=1. However, when comparing the FC quadrupole with the HT dipole contributions, these overlaps must also be considered.

To calculate the FC overlaps and the HT transition dipole moment of the $^1A_g^-$ state of all-trans beta-carotene we relied on TDDFT at the B3LYP/6-31G(d) level since previous works have shown that this choice can correctly describe the IR and Raman spectra of carotenoids as well as their vibrationally-resolved absorption spectra.[15,16] A common issue that one encounters when trying to calculate FC factors and HT terms of large molecules with flexible chemical groups is that, typically, the harmonic approximation fails. A straightforward way of checking this is to compare the reorganization energies calculated with the harmonic vibrational frequencies and the Huang-Rhys factors with those directly obtained from the adiabatic and vertical transition energies. In our case, the harmonic approximation failed because of low-energy vibrational frequencies that are highly anharmonic. By repeating the calculation for all-trans beta carotene and excluding low energy vibrational modes (<50 cm^{-1} and <160 cm^{-1}), the difference between the reorganization energies calculated via Huang-Rhys factors and adiabatic/vertical energies was reduced but still off by 0.10 eV. We therefore decided to test two model systems for all-trans beta-carotene. In the first model the terminal rings were frozen, and the second one only consisted of the polyene

backbone, including the four central methyl groups and without the terminal rings. The harmonic approximation holds for these two models, and they also reproduce quite faithfully the IR spectrum of all-trans beta-carotene, indicating that they capture the relevant vibrational modes and thus are suitable to calculate the FC factors and the HT contributions. Very similar results were obtained for both models and so, in the following, we report results based on the polyene backbone model.

In Figure S8 we compare the transition FC quadrupole and HT dipole moments of the $^1A_g^-$ excited state as a function of the vibronic transition frequency. In particular, we plot the cumulative value of these quantities, that is, we consider the contribution from all the vibronic transitions with frequency up to ω_k. The explicit expression for the FC transition quadrupole moment is

$$|Q(\omega_k)|^2 = Q_{el}^2 \sum_{t}^{k} \left| \left\langle \chi_{g,\vec{v}_i} \middle| \chi_{e,\vec{v}_f} \right\rangle \right|^2 (\omega_t)$$

where we have taken $Q_{el} = 0.1$ e·nm^2 as the electronic contribution (see Table S1). For the HT transition dipole moment, we have

$$|p(\omega_k)|^2 = \sum_{\alpha=x,y,z} \sum_{t}^{k} \sum_{m=1}^{N} \left(\frac{\partial p_{el,\alpha}}{\partial X_{m,\alpha}} \right)_{eq}^2 \left| \left\langle \chi_{g,\vec{v}_i} \middle| X_{m,\alpha} \middle| \chi_{e,\vec{v}_f} \right\rangle \right|^2 (\omega_t)$$

which directly follows from the expression we discussed above for the HT term of $\langle \Psi_g | \boldsymbol{p} | \Psi_e \rangle$. The shaded grey area in Figure S8 represents the width of the gaussian function that, in accordance with the experimental data in the main text, is used to model the β-carotene PL spectrum in the electromagnetic (EM) simulations (see next section). According to our calculations, considering the width of the PL band, i.e., the vibrations that contribute to the emission, the ratio between the FC quadrupole and the HT dipole is ~10 nm. From the results shown in Figure S8 we estimate the transition moments Q=0.06 e·nm^2 and p=0.006 e·nm (red dots in Figure S8) to be used in the main text and in the following section on the EM calculations. Note that Figure S8 plots results for absorption which are practically identical to those calculated for the emission process. Also note that, as expected (*vide supra*), the FC quadrupole moment converges to the electronic value of 0.1 e·nm^2 when the contribution from all vibronic transitions is considered.

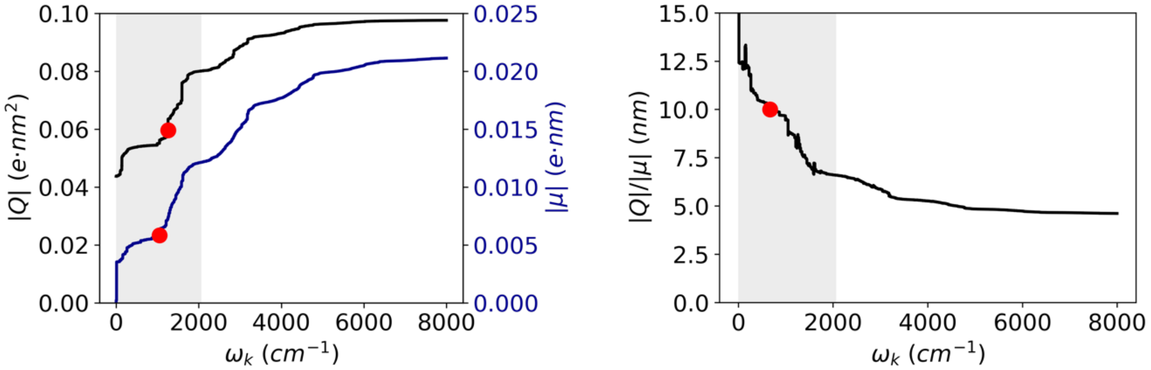

Figure S8. Calculated (cumulative) transition FC quadrupole (Q) and HT dipole (p) moments of β-carotene as a function of vibronic transition frequency for the $^1A_g^-$ excited state of beta-carotene. Red points indicate our estimate for the transition moments necessary for the EM simulations.

Some clarification is appropriate regarding the estimation of the transition dipole moment of the $^1A_g^-$ excited from experimental data available in the literature. In an exhaustive investigation with steady-state and time-resolved experiments, Gillbro and coworkers reported the radiative lifetime of the bright S_2 and dark S_1 states to be $\tau_{rad,2}\sim$ 1 ns and $\tau_{rad,1}\sim$ 2 μs, respectively,[17] implying the S_0-S_1 dipole transition to be more than 2000 times weaker than that of S_0-S_2.[18] This estimate is larger than the one we obtain from electronic structure calculations. A quick estimate considering the reported 2 μs radiative lifetime and the 0-0 transition frequency ~14300 cm^{-1} (~700 nm) leads to a transition dipole moment of the dark S_1 state ~0.75 D (=0.295 au = 0.015 e·nm). Note that this value implicitly contains the contribution from all the vibronic transitions and is close to the value we obtain in Figure S8 (~0.02 e·nm) for the limit of large ω_k.

For the classical EM simulations, the relative orientation between the FC quadrupole and the HT dipole moment of the $^1A_g^-$ excited state is required. To estimate the quadrupole moment, we diagonalized the electronic transition quadrupole tensor and took as its direction the eigenvector corresponding to the largest eigenvalue. For all the cases reported in Table S1, we found that the quadrupole tensor in the molecular frame centered at the center of nuclear charge (Figure S9) was already close to diagonal, with the largest element being Q_{xx}. As for the HT transition dipole, we calculated the $^1A_g^-$ excited state for the backbone model (including the methyl groups and excluding the terminal rings) and distorted the molecular geometry along the normal modes yielding the largest HT contributions. We found that the vibrationally-enabled transition dipole lied always along the polyene backbone, just like the transition quadrupole moment, as well as the transition dipole moment of the bright $^1B_u^+$ state (Figure S9). This observation is consistent with steady state fluorescence anisotropy experiments concluding that the transition dipole moment of the bright $^1B_u^+$ state is parallel to that of the $^1A_g^-$ state and aligned along the polyene chain.[17]

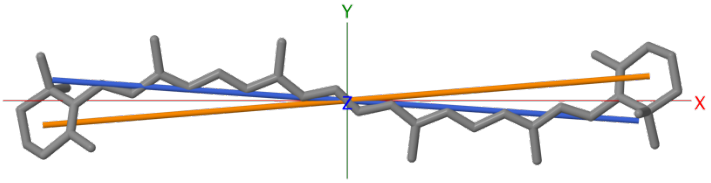

Figure S9. Electronic transition dipole moment of the $^1B_u^+$ state (blue) and quadrupole moment of the $^1A_g^-$ state (orange) of all-trans beta-carotene calculated at the TDDFT B3LYP/6-31G(d) level.

Electromagnetic simulations: Absorption and Purcell factor calculations

In this section, we provide details of the EM calculations performed to assess the contribution of the HT dipole and FC quadrupole moments to the dark transition between S_0 and S_1 states of the β-carotene molecules at the NPoM gap. All the simulations were performed with the finite-element, frequency-domain solver of Maxwell's Equations implemented in Comsol Multiphysics, and the convergence against mesh size and simulation volume was enforced in all cases.

First, we consider the absorption enhancement experienced by the molecules thanks to the plasmonic resonances sustained by the nanocavity under grazing (dark-field-like) plane wave illumination. The enhancement factor is defined as[19]

$$\frac{\gamma_{\mathrm{p}}^{\mathrm{abs}}}{\gamma_{\mathrm{p}}^{\mathrm{abs,0}}} = \frac{|\mathbf{p}\cdot\boldsymbol{E}|^2}{|\mathbf{p}|^2 E_0^2} = \left|\frac{E_\parallel}{E_0}\right|^2,$$

for a purely dipolar transition, and

$$\frac{\gamma_{\mathrm{Q}}^{\mathrm{abs}}}{\gamma_{\mathrm{Q}}^{\mathrm{abs,0}}} = \frac{|(\mathbf{Q}\boldsymbol{\nabla})\cdot\boldsymbol{E}|^2}{|\mathbf{Q}|^2 k_0^2 E_0^2} = \left|\frac{\partial_\parallel E_\parallel}{k_0 E_0}\right|^2,$$

for a purely quadrupolar one.[19] Note that we are taking free space as the reference, with an optimum dipole and quadrupole orientation (maximizing light-matter coupling). In the expressions above, $\boldsymbol{E}$ is the total electric field at the position of the molecule (obtained numerically) and E_0 is the amplitude of the plane-wave illumination).

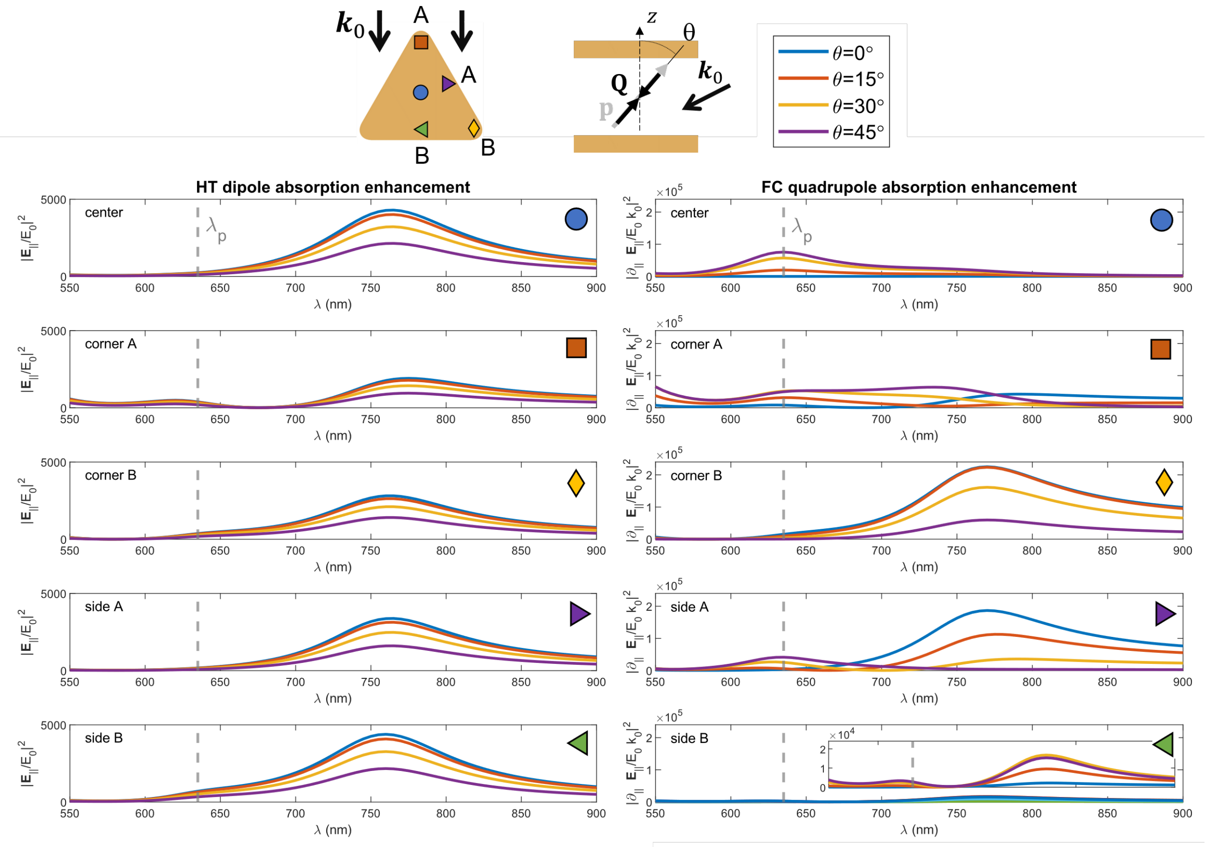

Figure S10. Absorption enhancement spectra for HT dipole (left) and FC quadrupole (right) transitions. The panels correspond to different molecule positions and include spectra for four different β-carotene orientations. At the top, the emitter positions and orientations are sketched (see also Table S2). Vertical dashed lines indicate the experimental pumping wavelength, $\lambda_p = 637$ nm.

Fig. S10 shows the absorption enhancement spectra for HT dipole (left) and FC quadrupole (right) moments placed within the middle plane of the NPoM gap and at different positions within the triangular-shaped section of the rhombicuboctahedral bottom facet. Each panel correspond to a different position, indicated by shaped (circular, square, rhombus, right- and left-oriented triangle) dots. In each panel, four different molecular orientations within the incidence plane are considered: 0º (vertical, blue), 15º (orange), 30º (yellow) and 45º (violet). Vertical dashed lines in all panels indicate the pumping wavelength, $\lambda_p = 637$ nm. Note that, in accordance with the electronic structure calculations (see previous section), the dipole and quadrupole moments are parallel to the β-carotene axis. The spectra present maxima originating from the plasmonic modes discussed in the main text: (10) at $\lambda \approx 630$ nm, and (20) at $\lambda \approx 770$ nm (see Fig. 1, high collection angle). Note that the signature of (11) mode cannot be resolved in the absorption spectra, due to its large overlapping with the (10) mode. The maximum absorption enhancement experienced by the FC quadrupole is approximately 2 orders of magnitude larger than the HT dipole.

Molecule position	*x* (nm)	*y* (nm)
center	0	0
corner A	0	27
corner B	23	-14
side A	7	12
side B	0	-14

Table S2. Molecule positions within the middle plane of the nanocavity gap ($z = d/2 = 1.2\,\mathrm{nm}$) considered in the EM calculations.

Next, we focus on the radiative Purcell enhancement experienced by the molecules in the NPoM gap. This is defined as[20]

$$\mathrm{P_p}(\lambda) = \frac{\kappa_\mathrm{p}^\mathrm{rad}}{\kappa_\mathrm{p}^\mathrm{rad,0}} = \frac{2\lambda}{\pi c\hbar\kappa_\mathrm{p}^\mathrm{rad,0}}\int_\Omega \langle \boldsymbol{S}_\mathrm{p}(\lambda)\rangle \cdot d\boldsymbol{s},$$

where$\kappa_\mathrm{p}^\mathrm{rad,0} = \kappa_\mathrm{p}^\mathrm{rad,0}(\lambda) = 8\pi^2|\mathbf{p}|^2/3\lambda^3\varepsilon_0\hbar$, $\langle \boldsymbol{S}_\mathrm{p}(\lambda)\rangle$ is the time-averaged Poynting vector generated by the dipolar source $\mathbf{p}$ placed at the NPoM gap (obtained numerically), and Ω is the far-field surface that accounts for the dark-field collection angle ($NA \approx 1$ in our calculations, mimicking the experimental θ_high). Similarly, the quadrupolar counterpart reads[21]

$$\mathrm{P_Q}(\lambda) = \frac{\kappa_\mathrm{Q}^\mathrm{rad}}{\kappa_\mathrm{Q}^\mathrm{rad,0}} = \frac{2\lambda}{\pi c\hbar\kappa_\mathrm{Q}^\mathrm{rad,0}}\int_\Omega \langle \boldsymbol{S}_\mathrm{Q}(\lambda)\rangle \cdot d\boldsymbol{s},$$

where $\kappa_\mathrm{p}^\mathrm{rad,0} = \kappa_\mathrm{p}^\mathrm{rad,0}(\lambda) = 4\pi^4|\mathbf{Q}|^2/45\lambda^5\varepsilon_0\hbar$ and $\langle \boldsymbol{S}_\mathrm{Q}(\lambda)\rangle$ is the numerical, time averaged Poynting vector generated by the quadrupolar source $\mathbf{Q}$ at the NPoM gap.

Fig. S11 presents the radiative Purcell enhancement spectra for HT dipole (left) and FC quadrupole (right) moments placed within the middle plane of the NPoM gap and at different positions within the triangular-shaped NPoM facet. It follows the same structure and labelling as Fig. S10. Again, the signatures of the plasmonic modes above are apparent in the spectra, and the maximum quadrupole Purcell factor is roughly 2 orders of magnitude larger than in the dipole case.

Grey dashed lines in all panels plot a gaussian model for the β-carotene PL spectrum, scaled to facilitate its visibility,

$$\mathcal{L}_{PL}(\lambda) = \frac{e^{(\lambda-\lambda_{PL})^2/2\Delta\lambda^2}}{\Delta\lambda\sqrt{2\pi}},$$

where, in accordance with the experiments, we have taken $\lambda_{PL} = 700\ \mathrm{nm}$ and $\Delta\lambda = 20\ \mathrm{nm}$. Note that the spectrum is normalized so that $\int \mathcal{L}_{PL}(\lambda)d\lambda = 1$.

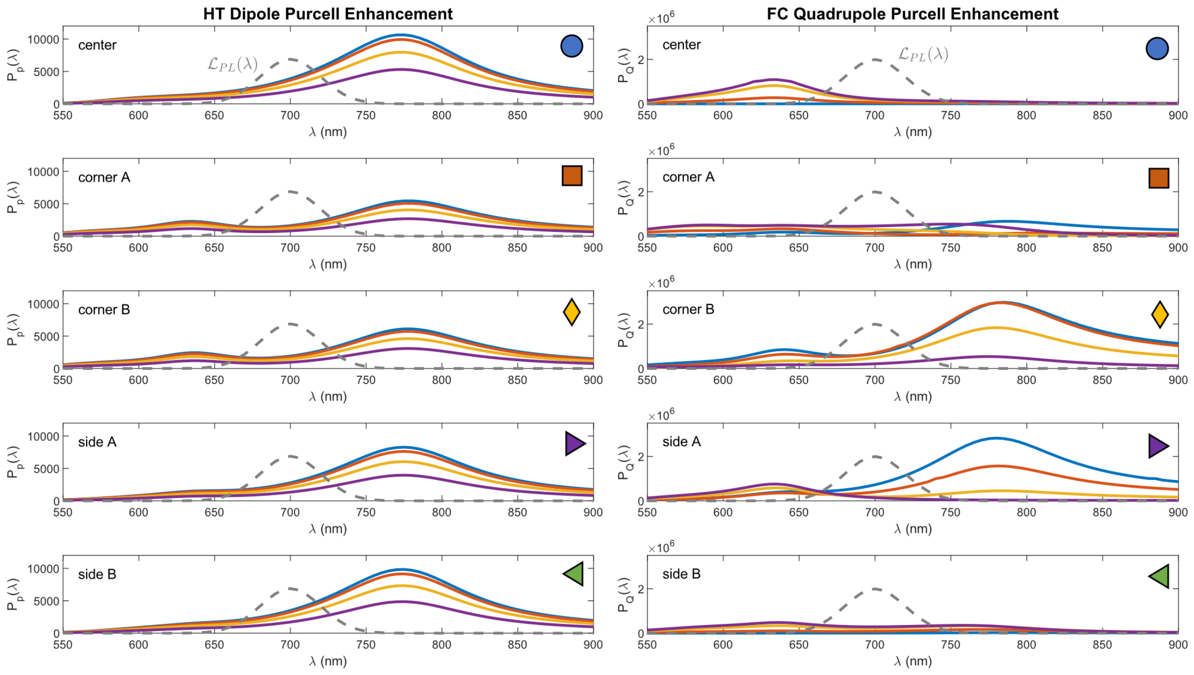

Figure S11. Radiative Purcell enhancement spectra for HT dipole (left) and FC quadrupole (right) transitions. The panels correspond to different molecule positions across the NPoM gap and include spectra for four different β-carotene orientations. The labels are the same as in Fig. S10. Grey dashed lines plot the gaussian profile used to model the molecular PL spectrum in free space (scaled for visibility).

With the magnitudes introduced above, the ratio between absorption and radiative rates for the HT dipole and FC quadrupole can be calculated as

$$\frac{\gamma_{\mathrm{p}}^{\mathrm{abs}}}{\gamma_{\mathrm{Q}}^{\mathrm{abs}}} = \left(\frac{|\mathbf{p} \cdot \boldsymbol{E}|^2}{|(\mathbf{Q}\boldsymbol{\nabla}) \cdot \boldsymbol{E}|^2} \right)_{\lambda=\lambda_p} = \left(\frac{\mathrm{p}^2 E_{\parallel}^2}{\mathrm{Q}^2 (\partial_{\parallel} E_{\parallel})^2} \right)_{\lambda=\lambda_p},$$

where $E_{\parallel}$ is the electric field component caused by the incident plane-wave at the β-carotene position and parallel to its axis, evaluated at the pumping wavelength, λ_p. Similarly, we can compute the ratio between radiative rates as

$$\frac{\gamma_{\mathrm{p}}^{\mathrm{rad}}}{\gamma_{\mathrm{Q}}^{\mathrm{rad}}} = \frac{\int \kappa_{\mathrm{p}}^{\mathrm{rad}}(\lambda)\, \mathcal{L}_{PL}(\lambda) d\lambda}{\int \kappa_{\mathrm{Q}}^{\mathrm{rad}}(\lambda)\, \mathcal{L}_{PL}(\lambda) d\lambda} = \frac{\int \kappa_{\mathrm{p}}^{\mathrm{rad},0}(\lambda) \mathrm{P}_{\mathrm{p}}(\lambda)\, \mathcal{L}_{PL}(\lambda) d\lambda}{\int \kappa_{\mathrm{Q}}^{\mathrm{rad},0}(\lambda) \mathrm{P}_{\mathrm{Q}}(\lambda)\, \mathcal{L}_{PL}(\lambda) d\lambda}.$$

Figure 4 in the main text presents the absorption and radiative enhancement spectra at the facet center, and an orientation tilted 30º with respect to the vertical direction. It also shows the two dipole-to-quadrupole ratios above for different molecule positions and orientations. This figure shows that both contributions are similarly relevant in absorption, while the HT dipole dominates radiation into the far-field.

Here, and to provide an overall picture of the relative weight of the HT dipole and FC quadrupole contributions to the $S_0 \longrightarrow S_1$ absorption of the β-carotene in the NPoM cavities treated as

completely independent, we consider next the PL ratio, defined as the product of the absorption and radiation decay rates above,[22]

$$\frac{\Gamma_{\rm p}}{\Gamma_Q}=\frac{\gamma_{\rm p}^{\rm abs}}{\gamma_{\rm Q}^{\rm abs}}\cdot\frac{\gamma_{\rm p}^{\rm rad}}{\gamma_{\rm Q}^{\rm rad}}=\left(\frac{{\rm p}^2E_\parallel^2}{{\rm Q}^2(\partial_\parallel E_\parallel)^2}\right)_{\lambda=\lambda_p}\frac{\int\kappa_{\rm p}^{\rm rad,0}(\lambda){\rm P}_{\rm p}(\lambda)\,\mathcal{L}_{PL}(\lambda)d\lambda}{\int\kappa_{\rm Q}^{\rm rad,0}(\lambda){\rm P}_{\rm Q}(\lambda)\,\mathcal{L}_{PL}(\lambda)d\lambda}.$$

As a reference, in free-space, this quantity can be computed approximately by neglecting dispersion effects, having (see main text)

$$\frac{\Gamma_{\rm p}^0}{\Gamma_{\rm Q}^0}\approx\left(\frac{|\mathbf{p}|^2}{k_0^2|\mathbf{Q}|^2}\right)_{\lambda=\lambda_p}\cdot\left(\frac{120|\mathbf{p}|^2}{k_0^2|\mathbf{Q}|^2}\right)_{\lambda=\lambda_{PL}}=1.8\cdot10^6.$$

where we have taken ${\rm p}=0.006\ {\rm e}\cdot{\rm nm}$ and ${\rm Q}=0.06\ {\rm e}\cdot{\rm nm}^2$ (see previous section in this SI).

Figure S12 plots the PL ratios at the nanocavity gap for different positions and orientations (see top insets of Fig. S10). The calculations assume that dipole and quadrupole PL mechanisms are completely independent, as if they corresponded to different emitters. This is not a valid approximation for our experimental samples, given that the absorption rates acquire comparable values (see main text) and therefore both channels become effectively coupled (see main text). Keeping this in mind, Fig. S12 indicates that the PL from vertical β-carotenes present a fully HT dipolar response. This is particularly apparent at the gap center, where $\frac{\Gamma_{\rm p}}{\Gamma_Q}\approx10^8$, and the nanocavity effectively increases the contrast between the PL rates with respect to free space (see above). On the contrary, for significantly tilted molecules, as expected for those in the experimental samples, PL rates approach each other, having $\frac{\Gamma_{\rm p}}{\Gamma_Q}\approx10$ for $\theta=45^{\rm o}$ at the facet center and positions at the gap edges that are directly illuminated by the incident plane-wave. So, even when treated independently, the gap plasmons sustained by the NpoM nanocavity reduce the contrast between the PL rates in free space by 5 orders of magnitude. Black dashed line plots the ratio of the PL rates in free space, $\Gamma_{\rm p}^0/\Gamma_{\rm Q}^0$.

By taking an average over positions and orientations in Fig S12, we obtain

$$\left\langle\frac{\Gamma_{\rm p}}{\Gamma_{\rm Q}}\right\rangle_{\theta,{\rm pos}}=72,$$

which shows that, treated independently (again, not a valid approximation in the experimental samples), the PL from the HT dipole is significantly larger that the FC quadrupole also inside the NpoM cavity. Note, however, that the contrast between them is more than 4 orders of magnitude lower than in free space. Fig. 4 in the main text shows though that the FC quadrupole is responsible for the main mechanism of optical absorption for β-carotenes at the NPoM gap that are tilted with respect to the vertical direction. This is at the origin of the enhanced PL emission observed experimentally.

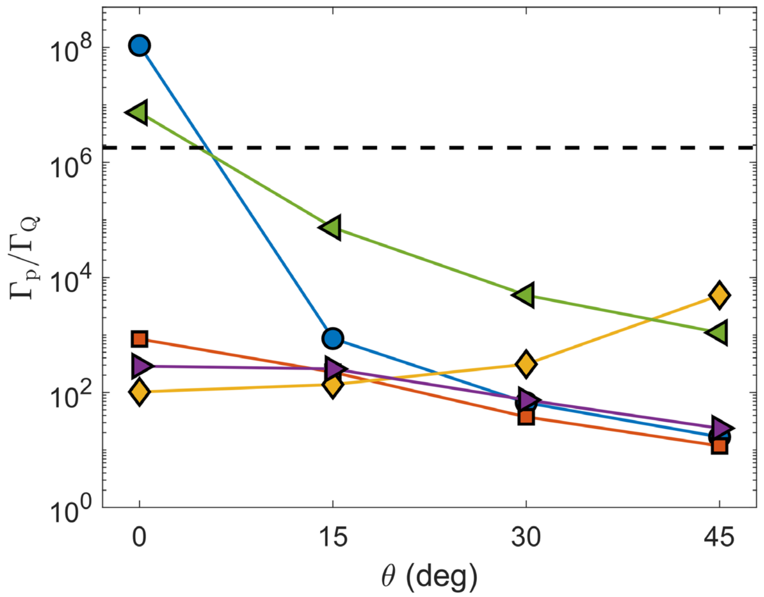

Figure S12. Ratio between HT dipole and FC quadrupole PL emission rates for molecules at different locations (different dot shapes, see top inset of Fig. S10), at the NPoM gap and for different orientations θ. Black dashed line plots $\Gamma_{\mathrm{p}}^{0}/\Gamma_{\mathrm{Q}}^{0} = 1.8 \cdot 10^{6}$ (free space, see above and main text).

Raman spectra of all-trans-β-carotene: experiment versus calculations

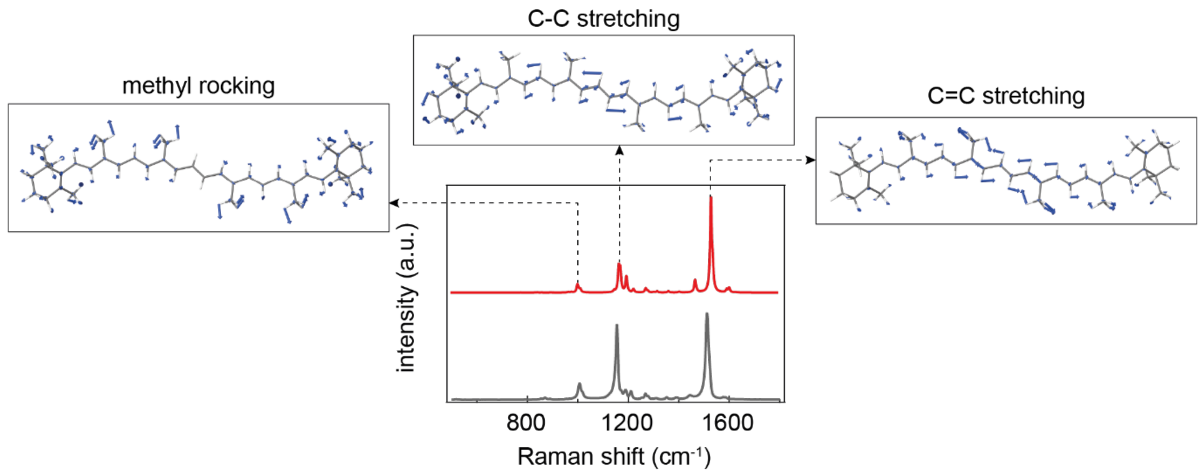

Fig. S13. Experimental (grey) and DFT calculated (red) Raman spectrum for *all-trans-β*-carotene powder with 3 dominant molecular vibrations depicted.

References

(1) Baró, A. M.; Hla, S.-W.; Rieder, K. H. LT-STM Study of Self-Organization of β-Carotene Molecular Layers on Cu (111). *Chemical Physics Letters* **2003**, *369* (1), 240–247.

(2) Epifanovsky, E. *et al.* Software for the Frontiers of Quantum Chemistry: An Overview of Developments in the Q-Chem 5 Package. *The Journal of Chemical Physics* **2021**, *155* (8), 084801.

(3) Chai, J.-D.; Head-Gordon, M. Long-Range Corrected Hybrid Density Functionals with Damped Atom–Atom Dispersion Corrections. *Phys. Chem. Chem. Phys.* **2008**, *10* (44), 6615–6620.

(4) Yanai, T.; Tew, D. P.; Handy, N. C. A New Hybrid Exchange–Correlation Functional Using the Coulomb-Attenuating Method (CAM-B3LYP). *Chemical Physics Letters* **2004**, *393* (1), 51–57.

(5) Cerón-Carrasco, J. P.; Requena, A.; Marian, C. M. Theoretical Study of the Low-Lying Excited States of β-Carotene Isomers by a Multireference Configuration Interaction Method. *Chemical Physics* **2010**, *373* (1), 98–103.

(6) Fiedor, L.; Dudkowiak, A.; Pilch, M. The Origin of the Dark S1 State in Carotenoids: A Comprehensive Model. *Journal of The Royal Society Interface* **2019**, *16* (158), 20190191.

(7) Starcke, J. H.; Wormit, M.; Schirmer, J.; Dreuw, A. How Much Double Excitation Character Do the Lowest Excited States of Linear Polyenes Have? *Chemical Physics* **2006**, *329* (1), 39–49.

(8) Hsu, C.-P.; Hirata, S.; Head-Gordon, M. Excitation Energies from Time-Dependent Density Functional Theory for Linear Polyene Oligomers: Butadiene to Decapentaene. *J. Phys. Chem. A* **2001**, *105* (2), 451–458.

(9) Andreussi, O.; Knecht, S.; Marian, C. M.; Kongsted, J.; Mennucci, B. Carotenoids and Light-Harvesting: From DFT/MRCI to the Tamm–Dancoff Approximation. *J. Chem. Theory Comput.* **2015**, *11* (2), 655–666.

(10) Szabo, A.; Ostlund, N. S. *Modern Quantum Chemistry: Introduction to Advanced Electronic Structure Theory*; Courier Corporation, 2012.

(11) Klessinger, M. & Michl, J. Excited States and Photochemistry of Organic Molecules (VCH, 1995).

(12) Frisch, M. J., Trucks, G. W., Schlegel, H. B., Scuseria, G. E., Robb, M. A., Cheeseman, J. R., Scalmani, G., Barone, V., Petersson, G., Nakatsuji, H. & others, Gaussian 16, Revision B.01 (2016).

(13) Duschinsky, F. On the Interpretation of Electronic Spectra of Polyatomic Molecules. *Acta Physicochim. URSS* **1937**, *7*, 551.

(14) Barone, V.; Bloino, J.; Biczysko, M. Vibrationally-Resolved Electronic Spectra in GAUSSIAN 09. *Revision a* **2009**, *2*, 1–20.

(15) Requena, A.; Cerón-Carrasco, J. P.; Bastida, A.; Zúñiga, J.; Miguel, B. A Density Functional Theory Study of the Structure and Vibrational Spectra of β-Carotene, Capsanthin, and Capsorubin. *J. Phys. Chem. A* **2008**, *112* (21), 4815–4825.

(16) Cerezo, J.; Zúñiga, J.; Requena, A.; Ávila Ferrer, F. J.; Santoro, F. Harmonic Models in Cartesian and Internal Coordinates to Simulate the Absorption Spectra of Carotenoids at Finite Temperatures. *J. Chem. Theory Comput.* **2013**, *9* (11), 4947–4958.

(17) Andersson, P. O.; Bachilo, S. M.; Chen, R.-L.; Gillbro, T. Solvent and Temperature Effects on Dual Fluorescence in a Series of Carotenes. Energy Gap Dependence of the Internal Conversion Rate. *J. Phys. Chem.* **1995**, *99* (44), 16199–16209.
(18) Bachilo, S. M.; Gillbro, T. Beta-Carotene S1 Fluorescence. In *5th International Conference on Laser Applications in Life Sciences*; SPIE, 1995; Vol. 2370, pp 719–723.
(19) Giannini, V.; Fernández-Domínguez, A. I.; Heck, S. C.; Maier, S. A. Plasmonic Nanoantennas: Fundamentals and Their Use in Controlling the Radiative Properties of Nanoemitters. *Chem. Rev.* **2011**, *111* (6), 3888–3912.
(20) Liu, M.; Lee, T.-W.; Gray, S. K.; Guyot-Sionnest, P.; Pelton, M. Excitation of Dark Plasmons in Metal Nanoparticles by a Localized Emitter. *Phys. Rev. Lett.* **2009**, *102* (10), 107401.
(21) Cuartero-González, A.; Fernández-Domínguez, A. I. Dipolar and Quadrupolar Excitons Coupled to a Nanoparticle-on-Mirror Cavity. *Phys. Rev. B* **2020**, *101* (3), 035403.
(22) Bharadwaj, P.; Deutsch, B.; Novotny, L. Optical Antennas. *Adv. Opt. Photon., AOP* **2009**, *1* (3), 438–483.
